# The Nation's Health

**Published:** 1923-09

**Authors:** 


### THE NATION'S HEALTH.

### SANITARY CONGRESS AT HULL.

THE Royal Sanitary Institute this year held its Annual Conference at Hull, under the presidency of the Right Hon. T. R. Ferens, High Steward of that city. An attractive programme, covering every phase of effort for the promotion of the public health, occupied the first week in August, and papers were read by distinguished authorities upon a wide variety of subject.

### The Need of the Moment.

In his presidential address Mr. Ferens pointed out that the greatest need of the nation at this moment is more houses, and said that it was estimated that the city of Hull itself needed approximately 6,000 more dwellings:—

In Hull, since 1899, they had been able to deal with over 1,400 unhealthy dwellings, which occupied sites aggregating a little more than eleven acres. He mentioned that in East Hull there was a well-laid-out garden village covering an area of 60 acres, and containing 600 houses, or ten to the acre. Every house had its bath and garden, and a marked improvement had occurred in the health of the inhabitants. Having alluded to the social and hygienic work in connection with a factory, also in East Hull, where over 5,000 workers of both sexes were employed, the president continued that poor health could rarely be attributed to working conditions in the factory; it was due rather to home conditions (drink and poverty), bad food, personal neglect and unsuitable clothing, especially boots.

### Detection of Tuberculosis.

A paper on "Methods for Detecting Tuberculosis in Workers Over the Age of Sixteen" was read by Dr. Raeburn, the Tuberculosis Medical Officer of Health:—

The death-rate from tuberculosis steadily declined after anti-tuberculosis machinery was introduced up till 1920. Since then it has remained stationary, and as half of the deaths from tuberculous disease are not notified till death or within three months of death, a large number of tuberculous cases are going about and infecting others. This, he thinks, is the probable explanation of the death-rate not declining further. The cause of undetected cases is the large number of open cases living uncontrolled amongst the general population. Infants and school children are systematically inspected by child welfare workers and school medical inspectors, and few cases of disease are missed by them. After leaving school, detection of disease is left to chance, and persons are usually far too advanced for effective treatment before they consult their doctor. Workers ought to be systematically examined for signs of early disease.

### Cancer Prevention.

In the course of a discussion on cancer a paper was read by Dr. A. Theodore Brand, of Driffield Poor Law Infirmary, in which he made suggestions for prevention:—

The burial of victims should be superseded by cremation. personal contact with the diseased should be avoided, flies should be killed on sight. If there were any truth in the allegation that the earthworm is an intermediate host of the cancer parasite, and that the soil is one habitat of it, then the mere idea of eating raw vegetables, however well scrubbed, and apparently clean, becomes repulsive. He recommended "segregation as in the case of leprosy." Cancer was not infectious in the ordinary sense; but it was transmissible from one individual to another.

### Ministry of Health as Fuel Expert.

Professor J. B. Cohen, of the University of Leeds, read a paper, with lantern illustrations, chiefly from Leeds, upon "The Smoke Evil":—

Any future legislation (he said) ought to be based on the principle that, where black smoke could be suppressed, it ought to be, and if it were not, there should be substantial penalties. The question was who was to say whether it could be suppressed. It seemed obvious to him that the only proper course was to have a fuel expert at the Ministry of Health and scientifically educated sanitary or smoke inspectors who would know where black smoke was a necessary accompaniment of the process, as it was in certain iron and steel works. He thought the Association of Manufacturers would be well advised to have their own expert to advise them and to appeal, where necessary, on their behalf to the fuel expert at the Ministry of Health. That was practically the system long since adopted in Germany, and let them look at the condition of manufacturing towns there.

### Should Unfit Babies be Preserved?

"Should Infantile Mortality be Reduced?" was the subject of a paper by Dr. F. E. Wynne, Medical Officer of Sheffield, which produced a lively discussion:—

If (he said) he were convinced that by preserving the lives of the hereditary degenerates and unfit they were deteriorating the moral and physical standard of the race, he would close every child-welfare centre in the country. By reducing infant mortality were they postponing the deaths of weakly children, he asked, or were they providing an environment in which good citizens would have a reasonable chance of surviving? He was entirely in agreement with the Eugenic School that the heredity of children should be studied, and that undesirable unions should be prevented. Finally, he said, he was convinced that in any case it was time that the thoughtless, sentimental propaganda in favour of the unlimited production of babies and their preservation, whether fit or unfit to survive, should be sternly discouraged by all who took public health work seriously.

### Port Sanitary Administration.

In the course of an address on this important subject, Alderman Askew said that the country owed an untold debt to the port authorities in preventing the admission and spread of infectious disease into the country:—

The sanitary organisation of the ports was such that not only was there an efficient medical service, but isolation hospitals were provided with the necessary nursing and administrative staffs for the purpose of forthwith isolating and treating any cases of infection as and whenever they were found. In addition there were disinfecting stations for contact, baths and a system of notification to distant medical officers of health of passengers or other persons who might be permitted to leave the port after the examination of ships. Alderman Askew also referred to the work of the examination of aliens, and the delicate and difficult duties of food inspection, and paid a high tribute to the work of the port sanitary officers generally, the performance of whose duties, he said, was invariably characterised by sound good sense, discrimination and judgment.

### Clean Food.

Dr. T. Eustace Hill, President of the Conference of Medical Officers of Health, appealed to the profession and the public to insist on cleaner and purer food. Generally speaking the mass of the population of this country, he said, are now provided with reasonably pure water, and, in consequence, there has been an extraordinary reduction in the prevalence of water-borne diseases, such as cholera and enteric fever. The importance of pure air is receiving increased attention, but in regard to articles of food very little has been done from the public health point of view to enforce purity, legislative action having been for the most part to prevent fraud rather than to safeguard health.

